# Metabolism-Based Therapeutic Strategies Targeting Cancer Stem Cells

**DOI:** 10.3389/fphar.2019.00203

**Published:** 2019-03-22

**Authors:** Petra Jagust, Beatriz de Luxán-Delgado, Beatriz Parejo-Alonso, Patricia Sancho

**Affiliations:** ^1^Centre for Stem Cells in Cancer and Ageing, Barts Cancer Institute, Queen Mary University of London, London, United Kingdom; ^2^Traslational Research Unit, Hospital Universitario Miguel Servet, Aragon Institute for Health Research (IIS Aragon), Zaragoza, Spain

**Keywords:** cancer stem cells, metabolism, mitochondria, oxidative phosphorylation, lipid metabolism, redox regulation

## Abstract

Cancer heterogeneity constitutes the major source of disease progression and therapy failure. Tumors comprise functionally diverse subpopulations, with cancer stem cells (CSCs) as the source of this heterogeneity. Since these cells bear *in vivo* tumorigenicity and metastatic potential, survive chemotherapy and drive relapse, its elimination may be the only way to achieve long-term survival in patients. Thanks to the great advances in the field over the last few years, we know now that cellular metabolism and stemness are highly intertwined in normal development and cancer. Indeed, CSCs show distinct metabolic features as compared with their more differentiated progenies, though their dominant metabolic phenotype varies across tumor entities, patients and even subclones within a tumor. Following initial works focused on glucose metabolism, current studies have unveiled particularities of CSC metabolism in terms of redox state, lipid metabolism and use of alternative fuels, such as amino acids or ketone bodies. In this review, we describe the different metabolic phenotypes attributed to CSCs with special focus on metabolism-based therapeutic strategies tested in preclinical and clinical settings.

## Introduction

Cancer is a highly heterogeneous disease, not only in terms of variability among patients, but also within a single tumor. This heterogeneity constitutes the main cause for therapy resistance and cancer progression in some patients (Hanahan and Weinberg, 2011). We can find different levels of intratumor heterogeneity. First, a tumor is comprised of multiple genotypes, which belong to distinct subclones with diverse features, which may include differential morphology and/or functionality. Additionally, tumors (and the subclones within) are formed of a functionally heterogeneous cell population, where a particular subset of tumor cells have the ability to initiate and propagate tumor growth, survive chemotherapy and drive relapse (Phillips et al., 2006; Diehn et al., 2009; Labelle et al., 2011; Hu et al., 2012; Rhim et al., 2012; Touil et al., 2014; Li et al., 2015b). These cells, the so-called cancer stem cells (CSCs), have self-renewal capacity, and can give rise to a differentiated progeny, leading to the production of all cell types present within a tumor, thereby generating tumor heterogeneity through a differentiation hierarchy (Reya et al., 2001). This distinct population was initially identified in leukemia, but was also found in solid tumors, such as breast, lung, prostate, colon, brain, head and neck, liver, as well as in pancreatic ductal adenocarcinoma (PDAC) (Hermann et al., 2007; Castellanos et al., 2013; Du et al., 2017). Finally, non-cancer cells present in the tumor microenvironment constitute a third level of heterogeneity, since they can directly affect cancer cell plasticity and functionality (Malanchi and Huelsken, 2009; Batlle and Clevers, 2017).

Cancer stem cells can be originated either from a mutation of a normal stem cell or from differentiated cells acquiring stem-like abilities (Reya et al., 2001). Indeed, numerous studies have found an abnormal activation of stem cell regulatory genes and pathways in the CSCs population, such as c-MYC, Bmi-1, Hedgehog, Notch and Wnt (Somervaille et al., 2009; Sancho et al., 2015; Wang et al., 2016). Apart from the well-known developmental pathways such as Wnt, Hedgehog or Jagged, metabolic traits have recently been involved in governing the function of stem cells. Indeed, although stem cells are primarily glycolytic, acquisition of certain metabolic plasticity, together with an increase in oxidative metabolism, primes them for maturation and supports their lineage differentiation (Cho et al., 2006; Chen et al., 2008; Simsek et al., 2010). A parallel mechanism was also postulated for CSCs in different tumors (Dong et al., 2013; Shen et al., 2015; Chen C.L. et al., 2016). However, recent data indicate that CSCs may mainly depend on oxidative metabolism (Sancho et al., 2015). In any case, reported metabolic differences between CSCs and progenies introduce another source of heterogeneity within tumors: metabolic heterogeneity. The latter can be further amplified, since different CSCs subclones can bear different metabolic phenotypes (Gammon et al., 2013) as a result of genetic or microenvironmental factors (Guha et al., 2014; Raj et al., 2015; Sancho et al., 2016).

## Cancer (Stem) Cell Metabolism

One of the main cancer characteristics is uncontrolled growth and cell division. To support the abnormal survival and growth, cancer cells need to increase nutrient uptake to supply biosynthesis pathways (Vander Heiden et al., 2009; Kamphorst et al., 2015; Hensley et al., 2016). To achieve that, cancer cells usually modulate the activity of different metabolic pathways in order to produce metabolic precursors to satisfy energetic and anabolic demands, and maintain redox balance (Vazquez et al., 2016). Due to the crucial contribution of diverse metabolic pathways to malignant transformation and tumor progression, metabolic reprogramming recently became one of the cancer hallmarks (Hanahan and Weinberg, 2011).

### Aerobic Glycolysis

The best example of reprogrammed metabolism in cancer is aerobic glycolysis (De Berardinis and Chandel, 2016): fast-proliferating tumor cells increase their glucose uptake to produce lactate in the presence of oxygen. This cancer hallmark was discovered by Otto Warburg and, thus, named the Warburg effect (Warburg et al., 1927; Warburg, 1956; Shim et al., 1998; Vander Heiden et al., 2009; Cairns et al., 2011). Glycolysis intermediates are used in diverse reactions to support high proliferation rates. For example, glucose-6-phosphate (G6P) can be used in the pentose phosphate pathway (PPP) to produce NADPH (Horton, 2002; Porporato et al., 2011; Doherty et al., 2014; Liberti and Locasale, 2016) or generate ribose groups, necessary for the synthesis of nucleotides (Lane and Fan, 2015; Vazquez et al., 2016). Alternatively, glycolytic intermediates can be used for anabolic reactions of glycogen or lipid synthesis (Gatenby and Gillies, 2004; Kroemer and Pouyssegur, 2008).

Glycolysis also facilitates survival and fast adaptation to the typically hypoxic tumor environment avoiding toxic Reactive Oxygen Species (ROS) accumulation through both low ROS production and increased detoxification systems (Brand and Hermfisse, 1997; Cairns et al., 2011; Doherty et al., 2014; Liberti and Locasale, 2016). Moreover, favoring glycolysis may bring other advantages to tumor cells, such as creating an acidic environment that can help invasion and suppress the immune response (Fischer et al., 2007; Swietach et al., 2007).

Even though aerobic glycolysis is quite inefficient in terms of ATP production, the rate of glucose uptake can be significantly elevated in cancer cells, resulting in ATP production to levels usually achievable with oxidative phosphorylation (OxPhos) (Liberti and Locasale, 2016). Additionally, although it was originally postulated that aerobic glycolysis is irreversible for tumor cells after cell division (Zu and Guppy, 2004; Jose et al., 2011; Porporato et al., 2011), it is well accepted nowadays that most cancers still produce ATP via OxPhos and modulate the contribution of both pathways in response to environmental factors or in different phases of the cell cycle (Smolková et al., 2011; De Berardinis and Chandel, 2016).

Importantly, glycolytic metabolism supports stemness in normal stem cells and CSCs of several cancer types (Folmes et al., 2011) (Table 1). Indeed, recent pieces of evidence demonstrate the involvement of oncogenes and pluripotency transcription factors, such as MYC, p53, K-Ras, HIF1α, NANOG, MEIS1, Wnt or OCT4 in the metabolic reprogramming from oxidative-dependent metabolism to a glucose dependence in many types of cancer (reviewed in Gabay et al., 2014; Alptekin et al., 2017; Deshmukh et al., 2018). Different studies support the glycolysis dependence of CSCs in diverse types of cancer, such as in radioresistant nasopharyngeal carcinoma spheres with high expression of stage-specific embryonic antigen (SSEA) -3 and -4 compared to parental cells (Shen et al., 2015), CD133^+^ human hepatocellular carcinoma cells and mouse models (Song et al., 2015; Chen C.L. et al., 2016), ALDH^+^ (aldehyde dehydrogenase) non-small lung carcinoma cells and side population (SP) cells from human colon cancer (Liu et al., 2014).

**Table 1:** Stem-like cells with glycolytic metabolism for various cancer types (in chronological order).

METABOLIC PHENOTYPE: GLYCOLYSIS				

Cancer type	Model of study	CSCs/Tumor cells	Methods	References
Glioblastoma	*In vivo* (xenograft) and *in vitro*	Neurospheres	Clark-type oxygen electrode	Zhou et al., 2011
Glioblastoma	*In vitro*	Neurospheres	Gene expression analysis	Goidts et al., 2012
Breast cancer	*In vitro*	Bulk of tumoral cells	Isotope tracing and seahorse	Dong et al., 2013
Glioblastoma	*In vitro*	Neurospheres	Clark-type oxygen electrode	Yuan et al., 2013
Ovarian cancer	*In vivo* (xenograft) and *in vitro*	Spheres	Isotope tracing and seahorse	Anderson et al., 2014
Breast cancer	*In vitro*	Spheres	Proteomics and targeted metabolomics	Ciavardelli et al., 2014
Ovarian cancer	*In vitro*	Spheres	Isotope tracing combined with spectrometry	Liao et al., 2014
Lung cancer	*In vitro*	SP	Clark-type oxygen electrode	Liu et al., 2014
Colorectal cancer	*In vitro*	SP	Clark-type oxygen electrode	Liu et al., 2014
Osteosarcoma	*In vitro*	3AB-OS CSC-like line	Seahorse	Palorini et al., 2014
Teratocarcinomas	*In vitro*	P19SCs	Clark-type oxygen electrode	Vega-Naredo et al., 2014
Nasopharyngeal carcinoma	*In vitro*	Sphere-derived cells	Seahorse	Shen et al., 2015
Hepatocellular carcinoma	*In vitro*	CD133^+^cells	Seahorse	Song et al., 2015
Lung cancer	*In vitro*	Spheres	Glucose uptake, glutamine, glutamate and NAD+/NADH determination	Deshmukh et al., 2018
Breast cancer	*In vitro*	Spheres	Glucose uptake, glutamine, glutamate and NAD+/NADH determination	Deshmukh et al., 2018
Brain cancer	*In vitro*	Tumor cell lines with BTIC features	Seahorse	Malchenko et al., 2018


On the other hand, enhanced glycolysis in CSCs could also constitute a secondary response to maintain energy balance, since reduction of mitochondrial metabolism seems to be essential for full stemness in some cancer types, such as osteosarcoma or glioblastoma (Zhou et al., 2011; Yuan et al., 2013; Palorini et al., 2014). Indeed, the downregulation of mitochondrial genes was associated with enhanced increased expression of genes related to epithelial-mesenchymal transition (EMT) usually linked to stemness (Gaude and Frezza, 2016). Importantly, such inverse relationship was functionally proven in embryonal carcinoma cells derived from teratocarcinomas, since the stimulation of mitochondrial function induced cell differentiation and loss of pluripotency (Vega-Naredo et al., 2014). In fact, the occurrence of this metabolic switch, not the final glycolytic phenotype, seems to be key for early state of tumorigenesis and acquisition of stemness-related properties in human mammospheres and brain CSCs in a mouse model of primitive neuroectodermal tumors (Dong et al., 2013; Ciavardelli et al., 2014; Malchenko et al., 2018).

### Mitochondrial Metabolism

Mitochondria play a key role in eukaryotic cells coordinating energy production and distribution through OxPhos based on oxygen and substrate availability, although other important metabolic reactions such as fatty acid oxidation (FAO), glutaminolysis, or reductive carboxylation in cells with damaged mitochondria also take place in these organelles. Mitochondrial tricarboxylic acid (TCA) cycle is primarily fueled by acetyl-CoA produced by glycolysis (from pyruvate) or FAO. Alternatively, in highly glycolytic cells, such as Ras-mutant cells, glutamine can be the driving force for OxPhos (Fan et al., 2013) through its conversion to α-ketoglutarate and oxaloacetate, that can be then used for fatty acids (FAs) and nucleotide synthesis (Gaglio et al., 2011). Electron donors produced in the TCA cycle are used by the electron transport chain (ETC) to create a proton motive force to synthesize ATP by the complex V.

As opposed to what we summarized in the previous section, recent literature described OxPhos as the main source of energy in CSCs from a number of cancer types (Table 2). This has been convincingly shown for ROS^low^ quiescent CD34^+^ leukemia CSCs (Lagadinou et al., 2013), lung spheroids and CD133^+^ PDAC cells (Ye et al., 2011; Sancho et al., 2015), as well as CSCs-enriched spheroids form ovarian, cervical and papillary thyroid carcinoma that displayed a reprogrammed metabolism through TCA cycle (Sato et al., 2016; Caria et al., 2018). Since mitochondrial metabolism coupled to OxPhos constitutes a much more efficient energy process, CSCs relying on OxPhos would theoretically make a better use of limited nutrients, which is an important advantage to survive in nutritionally poor environments. Indeed, mitochondria-dependent CD44^+^CD117^+^ ovarian CSCs and CD133^+^ PDAC CSCs showed enhanced resistance to glucose or glutamine deprivation compared to their differentiated counterparts (Pasto et al., 2014; Sancho et al., 2015). On the other hand, a variety of metabolites released by stromal cells can be used by OxPhos-dependent cells to fuel the TCA cycle, conferring them with increased adaptability to the changing conditions of the tumor microenvironment (Anderson et al., 2014). The best known example is lactate uptake from hypoxic tumor cells or cancer-associated fibroblasts (CAFs) via monocarboxylate transporter 1 and 2 (MCT1 and MCT2) in a process known as reverse Warburg effect (Kroemer and Pouyssegur, 2008; Sonveaux et al., 2008; Porporato et al., 2011; Rattigan et al., 2012). Moreover, pancreatic stellate cells release alanine to fuel the TCA cycle and subsequent biosynthetic pathways in pancreatic cancer cells (Sousa et al., 2016). Additionally, recent evidence indicate that microvesicles found in the tumor microenvironment contain several metabolites, including aminoacids, lipids and TCA cycle intermediates to fuel central metabolism of oxidative tumor cells and, consequently, tumor growth (Santi et al., 2015; Zhao et al., 2016). In this sense, stromal cells would play a key role in tumor progression supporting OxPhos-dependent CSCs proliferation and survival in nutrient-deprived environments.

**Table 2:** Stem-like cells with OxPhos metabolism for various cancer types (in chronological order).

METABOLIC PHENOTYPE: OxPhos				

Cancer type	Model of study	CSC/Tumor cells	Methods	References
Lung cancer	*In vivo* (xenograft) and *in vitro*	Secondary spheres	Clark-type oxygen electrode	Ye et al., 2011
Glioblastoma	*In vitro*	Gliomaspheres	Seahorse	Janiszewska et al., 2012
Leukemia stem cells	*In vitro*	CD34^+^ cells	Seahorse	Lagadinou et al., 2013
PDAC	*In vivo* (inducible mouse model of mutated KRAS2) and *in vitro*	Spheres	Isotope tracing, metabolomics and seahorse	Viale et al., 2014
Breast cancer	*In vitro*	Spheres	Label-free quantitative proteomics	Lamb et al., 2015b
PDAC	*In vivo* (xenograft) and *in vitro*	CD133^+^ cells and spheres CD44^+^CD133^+^	Seahorse	Sancho et al., 2015
Ovarian cancer	*In vitro*	Spheres	Metabolomics	Sato et al., 2016
Papillary Thyroid Carcinoma	*In vitro*	Thyrospheres	GCMS	Caria et al., 2018


Even though Warburg hypothesized that mitochondrial respiration defects are responsible for cancer cells shifting to glycolysis, it is known today that cancer cells still retain mitochondrial functions and that, in fact, a significant amount of ATP is produced through OxPhos (Tang et al., 2011; Kang et al., 2014; Zong et al., 2016). Indeed, ATP from OxPhos proved to be important for cell movements and invasive/metastatic abilities of cancer (stem) cells (Yu et al., 2017), suggesting that mitochondria contributes to cytoskeletal alterations (Caino et al., 2015). Moreover, OxPhos activation in metastatic breast cancer models is crucial to escape from the metabolic dormancy derived from hormonal therapy (Sansone et al., 2016). Interestingly, OxPhos activation is caused by the horizontal transfer of mitochondrial DNA (mtDNA) in exosomes from CAFs to dormant CSCs, providing a possible mechanism to development of resistance to hormonal therapy and highlighting metabolic interaction between CSCs and their niche (Sansone et al., 2017).

Beyond energy production, mitochondria are involved in controlling cellular redox rate, ROS generation, calcium buffering and the synthesis of intermediate molecules, such as acetyl-CoA and pyrimidines. Additionally, mitochondria have a crucial role in apoptosis initiation through activation of the membrane permeability transport pore, and release of cytochrome C (Wallace, 2012). Furthermore, mitochondria may contribute to malignant transformation and tumor progression through increased ROS production by the ETC (Ishikawa et al., 2008; Liou et al., 2016), abnormal accumulation of specific mitochondrial oncometabolites modifying epigenetic signals (Sciacovelli et al., 2013), and functional deficits in apoptosis (Tomiyama et al., 2006; Izzo et al., 2016). For all these reasons, mitochondrial biogenesis is essential for survival and propagation of CSCs regardless of their metabolic phenotype (Bonuccelli et al., 2010; De Luca et al., 2015; De Francesco et al., 2018). In fact, mitochondrial biogenesis may be a primary driver of stemness since its inhibition efficiently eliminated hypoxic spheroids in breast cancer (Lamb et al., 2015b,c; De Francesco et al., 2017).

The mechanisms driving mitochondrial biogenesis and OxPhos in CSCs described above have not been fully characterized yet, although some studies shed some light on this matter. In fact, findings in glioblastoma spheroids demonstrated the role of the oncofetal insulin-like growth factor 2 mRNA-binding protein 2 (Imp2) in the regulation of OxPhos, and mitochondrial biogenesis and structure (Janiszewska et al., 2012). Interestingly, the metabolic profile and plasticity of PDAC CD133^+^ cells rely on the balance between the MYC-driven glycolysis and the main regulator of the mitochondrial biogenesis peroxisome proliferator-activated receptor gamma coactivator 1-alpha (PGC-1α) (Sancho et al., 2015). In this study, differentiated PDAC cells exhibited an overexpression of MYC that counteracted stemness maintenance through a negative regulation of PGC-1α. These results apparently contradict the role of MYC as driver of stemness via glycolysis previously exposed, which may be due to a cell context-dependent modulation of stemness/differentiation. On the other hand, PGC-1α overexpression can also lead to different outcomes depending on the cellular context in BRAF driven melanomas: increased PGC-1α expression in primary tumors after BRAF inhibition with vemurafenib causes OxPhos addiction associated with poor patient prognosis (Haq et al., 2013), while it impaired growth rate and invasive abilities in metastatic settings (Luo et al., 2016).

### Redox Regulation

It is well known that oncogenic transformation (Trachootham et al., 2009; Zhou et al., 2014), dysfunctional mitochondria (Kudryavtseva et al., 2016) and altered cell signaling (Tachibana et al., 2008; Raza et al., 2017) induce ROS accumulation in cancer cells, further promoting tumorigenesis and mutagenesis. However, due to the potential deleterious effects of ROS, a powerful antioxidant machinery formed of both enzymatic and non-enzymatic antioxidants is often found in cancers (Obrador et al., 2002; Townsend and Tew, 2003; Arnold et al., 2004; Estrela et al., 2006; Valko et al., 2006; Du et al., 2013; Harris et al., 2015; Raza et al., 2017).

Increasing evidence suggests an important role of ROS and redox signaling for CSCs functionality. It was known that quiescent stem cells reside in a low ROS niche that supports their stemness characteristics, like self-renewal capacity. On the other hand, increased ROS content promote stem cell proliferation and differentiation (Ito et al., 2006; Jang and Sharkis, 2007; Naka et al., 2008; Owusu-Ansah and Banerjee, 2009; Yahata et al., 2011; Bigarella et al., 2014). Only recently, it was shown that CSCs share the same redox-related properties (Diehn et al., 2009; Kobayashi and Suda, 2012; Yuan et al., 2015): murine CD44^+^CD24^-/low^Lin^-^ and human Thy1^+^CD24^+^Lin^-^ breast CSCs (Diehn et al., 2009; Luo et al., 2018), CD44^high^ gastrointestinal cell lines (Ishimoto et al., 2011), tumorigenic ROS^low^ from head and neck carcinoma cell lines (Chang et al., 2018), human or murine CD133^+^ glioblastoma cells from cell lines and tumors and chronic myeloid leukemia (CML) CD34^+^ CSCs (Zhao et al., 2009; Qiang et al., 2012; Zhu et al., 2013) maintain low levels of intracellular ROS coupled to enhanced antioxidant capacity. Apart from stemness maintenance, bearing high antioxidant capacity grants CSCs resistance to ROS inducers, such as chemo and radiotherapy (Diehn et al., 2009; Izumiya et al., 2012; Wang et al., 2017b).

Redox balance can also be achieved through the regulation of ROS-dependent signaling pathways and redox-sensitive transcription factors, such as c-MYC, HIF1α, p53, NF-κB, AP-1, and the master regulator of antioxidant response, nuclear factor erythroid 2–related factor 2 (NRF2) (Chandel et al., 2000; Kamata et al., 2005; Soriano et al., 2009; Liou and Storz, 2010; Boyer-Guittaut et al., 2014; Jiang et al., 2014; Raza et al., 2017). In fact, CSCs regulate ROS levels via antioxidant transcription factors, such as NRF2 or FOXO (Diehn et al., 2009; Zhu et al., 2013; Wu T. et al., 2015; Ryoo et al., 2016; Chang et al., 2018; Luo et al., 2018). Most of these factors affect redox homeostasis by direct or indirect modulation of cellular metabolism. Indeed, NRF2 upregulation in different cancer types (Mitsuishi et al., 2012; Abdul-Aziz et al., 2015; Kim and Keum, 2016; Rocha et al., 2016; Milkovic et al., 2017) influences the switch between anabolic/catabolic glucose metabolism (Mitsuishi et al., 2012; Wallace, 2012; Heiss et al., 2013; Hawkins et al., 2016). In addition, the proto-oncogene c-MYC controls both cellular metabolism and redox homeostasis by increasing glycolysis (Ellwood-Yen et al., 2003; Kim et al., 2007; Wang et al., 2008; Miller et al., 2012; He et al., 2015; Davis-Yadley et al., 2016; Massihnia et al., 2017), and regulating glutamine metabolism (Anderton et al., 2017). Interestigly, both mechanisms could be interconnected, since c-MYC binds to the NRF2 promoter (Levy and Forman, 2010).

Importantly, most of the main signaling pathways governing CSCs functionality are regulated by ROS signaling. That is the case of stemness-regulatory pathways, such as Wnt and Notch (Takubo et al., 2010; Qiang et al., 2012; Paul et al., 2014), or key signaling nodes important for cell survival and growth, such as PTEN (Xia et al., 2013), PI3K (Le Belle et al., 2011), AKT (Zhou et al., 2007; Dey-Guha et al., 2011), ATM (Ito et al., 2004; Yalcin et al., 2008), STAT3 (Qiang et al., 2012; Zhang et al., 2016), and mammalian target of rapamycin (mTOR) (Dubrovska et al., 2009) and their downstream targets. Moreover, those pathways further modulate ROS production/detoxification in a positive feedback loop by activating redox-sensitive transcription factors (Miyamoto et al., 2007; Tothova et al., 2007; Dubrovska et al., 2009; Yeo et al., 2013; Zhang et al., 2016).

### Lipid Metabolism

Besides the classical metabolic reprogramming related to glucose, alterations in diverse aspects of lipid metabolism are increasingly gaining attention as determinants of cancer, including CSCs function. In fact, highly proliferating cells require increased amounts of the cell membrane’s main components: lipids and cholesterol. In that cellular location, lipids function as either membrane building blocks or signaling transduction modifiers, since membrane lipid composition modulates protein recruitment and interaction (lipid rafts) (Rysman et al., 2010; Staubach and Hanisch, 2011). In this sense, several reports indicate that CSCs accumulate unsaturated lipids, such as monounsaturated FAs (MUFAs), the precursors of several plasma membrane lipids. In fact, lipid desaturation, mainly via the enzyme stearoyl-CoA desaturase (SCD-1), plays essential functions controlling self-renewal and tumorigenicity in different cancer models (Noto et al., 2013, 2017; Lai et al., 2017; Li F. et al., 2017), possibly through the activation of stemness-related pathways, such as Wnt signaling (Lai et al., 2017). Additionally, differences in plasma membrane lipid composition between CSCs and their differentiated counterparts have been reported, which can be potentially used to identify CSCs. Indeed, even though CSCs may present an overall decrease in glycosphingolipids as described for the glioblastoma CSC-like cell line GSC11 (He et al., 2010), the expression of specific gangliosides, such as GD2 and GD3, identified cells with increased self-renewal capacity and tumorigenicity in breast cancer (Battula et al., 2012; Liang et al., 2013).

Lipids and cholesterol in tumors are either scavenged from exogenous sources or synthesized *de novo* through FA synthase (FASN) or the mevalonate pathway, respectively (Beloribi-Djefaflia et al., 2016). Thus, different reports suggest that elevated *de novo* synthesis of lipids and cholesterol contribute to CSCs properties and survival. In fact, the expression of sterol regulatory element-binding protein 1 (SREBP1), master controller of *de novo* lipogenesis, is increased in CD24^-^CD44^+^ESA^+^ cells from a ductal carcinoma *in situ* cell line as well as mammospheres and melanospheres (Pandey et al., 2013; Corominas-Faja et al., 2014; Giampietri et al., 2017). This transcription factor may be involved in resistance to hypoxia and nutrient scarce environments, as suggested for glioblastoma sphere-derived cells (Lewis et al., 2015). Moreover, *de novo* lipogenesis from glycolytic intermediates or acetate via FASN is critical for *in vitro* self-renewal (Corominas-Faja et al., 2014; Yasumoto et al., 2016), and tumor relapse and metastatic dissemination after withdrawal of anti-angiogenic treatment (Sounni et al., 2014). In the same line of evidence, the activation of the mevalonate pathway is important for self-renewal and tumor formation in breast and pancreatic cancer, as well as glioblastoma (Ginestier et al., 2012; Brandi et al., 2017; Wang et al., 2017a).

Although *de novo* synthesis has traditionally been considered the preferred source of FAs for tumor cells (Ookhtens et al., 1984), recent reports highlight the crucial role of FAs uptake via CD36 or FA binding proteins (Hale et al., 2014; Pascual et al., 2016). The same is also true for cholesterol uptake within lipoproteins (Guillaumond et al., 2015). Indeed, lipid uptake, either via lipoprotein receptors or CD36, favors proliferation of glioma CD133^+^ cells (Hale et al., 2014) and label-retaining/CD44^+^ cells from squamous cell carcinoma (Pascual et al., 2016). Interestingly, increased lipid uptake points to the crucial role of microenvironment supporting cancer (stem) cell functions: tumor-activated adipocytes provide FAs to support leukemia CD34^+^ cells growth, survival and chemoresistance (Ye et al., 2016; Shafat et al., 2017) as well as omental metastasis from ovarian cancer (Nieman et al., 2011).

Fatty acids require covalent modification by CoA by fatty acyl-CoA synthetases to enter the bioactive pool of FAs. Afterward, they will be further esterified to form triacylglycerols or sterol esters and stored in lipid droplets (LDs). Importantly, recent reports correlate accumulation of LDs or stored cholesteryl-ester with tumor progression and aggressiveness (Yue et al., 2014; Guillaumond et al., 2015). In fact, activated and stored lipids play a crucial role supporting tumorigenicity of CSCs *in vivo*, as demonstrated in cells derived from neurospheres from glioblastoma and ALDH^+^ CD133^+^ ovarian cancer cells (Sun et al., 2014; Menard et al., 2016; Li J. et al., 2017). This may be a reflect of adaption to the harsh conditions found in the tumor microenvironment, since those lipids can be mobilized upon metabolic stress, providing ATP via FAO to ensure survival (Maan et al., 2018). Importantly, increased lipid storage in LDs may constitute a useful CSCs marker, as demonstrated in colorectal (CRC) and ovarian cancer (Tirinato et al., 2015; Li J. et al., 2017).

Activated FAs are not only incorporated into membranes or storage, but also used as substrate to synthesize signaling lipids or energy production in FAO. Although FAO is considered the main energy source in non-glycolytic tumors (Liu et al., 2010; Caro et al., 2012), a high activity of this pathway has been reported for aggressive tumor cells and CSCs, especially in nutrient scarce environments (Table 3) (Carracedo et al., 2013; Kamphorst et al., 2013; Daniëls et al., 2014; Pasto et al., 2014). In fact, ATP production and survival of matrix-deprived epithelial cells depend on FAO (Schafer et al., 2009; Carracedo et al., 2012), a metabolic process that also sustains the self-renewal capacity in both leukemia-initiating CFSE^high^CD34^+^ cells and hematopoietic long-term culture initiating cells (Samudio et al., 2010; Ito et al., 2012). Besides its well-known role in energy production, FAs metabolism via mitochondrial FAO regulates multiple functions of CSCs. Indeed, FAO contributes to pluripotency maintenance and chemoresistance (Wang T. et al., 2018), mainly by reducing ROS production (Lee et al., 2015; Chen et al., 2016) and may sustain metastatic properties of sphere-derived cells (Aguilar et al., 2016).

**Table 3:** Stem-like cells using alternative metabolism for various cancer types (in chronological order).

METABOLIC PHENOTYPE: OTHERS					

Cancer type	Metabolic phenotype	Model of study	CSC/Tumor cells	Methods	References
Breast cancer	FAO	*In vitro*	Detached tumor cells	Isotope tracing	Schafer et al., 2009
Breast cancer	Ketone bodies	*In vivo* (xenograft)		3-OH-butirate effects on tumor growth, migration and angiogenesis	Bonuccelli et al., 2010
Hepatic cancer	Glutamine	*In vitro*	Bulk of tumor cells	BD Oxygen Biosensor System	Hu et al., 2010
Leukemia-initiating cells	FAO	*In vivo* (xenograft) *In vitro*	Bulk of tumor cells	Clark-type oxygen electrode	Samudio et al., 2010
Hepatic cancer	Glutamine	*In vitro*	Bulk of tumor cells	Glutathione, glutamate and glutamine	Suzuki et al., 2010
Breast cancer	FAO	*In vitro*	Detached tumor cells	Isotope tracing	Carracedo et al., 2012
Leukemia-initiating cells	FAO	*In vivo*	CD150^+^CD48^-^CD41^-^Flt3^-^CD34^-^KSL cells sorted from Pml^+^/^+^ or Pml^-^/^-^mice	Isotope tracing and seahorse	Ito et al., 2012
Glioblastoma	PPP	*In vitro*	Gliomaspheres	Isotope tracing	Kathagen et al., 2013
Colorectal cancer	Glycolysis, TCA cycle, and cysteine/methionine metabolism	*In vitro*	CD133^+^ cells	Metabolomics	Chen et al., 2014
Ovarian Cancer	OXPHOS and PPP	*In vivo* (xenografts) *In vitro*	CD44^+^CD117^+^ cells	Flow cytometry	Pasto et al., 2014
PDAC	Glutamine (non-canonical pathway of glutamine metabolism)	*In vivo* (xenografts) *In vitro*	Spheres	Gene expression and enzymatic assays	Li D. et al., 2015
Colorectal cancer	Lysine catabolism	*In vivo* (xenografts) *In vitro*	CD110^+^	Transcriptomics	Wu Z. et al., 2015
Hepatocellular carcinoma	Glycolysis and FAO in sh-Nanog-TICs	*In vitro*	CD133^+^CD49f^+^CD45^-^	Isotope tracing and metabolomics	Chen C.L. et al., 2016
Breast cancer	PPP	*In vitro*	Mammospheres and ALDH^+^ cells	Glucose consumption, lactate, NADPH and G6P	Debeb et al., 2016
Cervical cancer	TCA	*In vitro*	Spheres	Metabolomics	Sato et al., 2016
Breast cancer	Mitochondrial biogenesis and FAO	*In vitro*	Mammospheres	Seahorse and label-free semi-quantitative proteomics	De Francesco et al., 2017
Pancreatic cancer	Glutamine	*In vitro*	ABCG2 high	ATP, NADP+/NADPH and glutathione	J Liao et al., 2017
Breast cancer	Ketone bodies	*In vitro*	Mammospheres	Seahorse	Ozsvari et al., 2017
Brain cancer	Purine metabolism	*In vivo* (xenograft) and *In vitro*	Brain TICs	Metabolomics	Wang et al., 2017c


Finally, lipids can also regulate CSCs functionality in terms of self-renewal and tumorigenic abilities through their function as second messengers in signal transduction pathways, thus becoming potential therapeutic targets. Indeed, sphingolipids, such as sphingosine-1-P (S1P), eicosanoids, such as prostaglandin E2 or glycerophospholipids, such as lysophosphatidic acid (LPA), have been reported to increase CSCs proliferation and *in vivo* tumorigenicity, activating self-renewal and survival signaling pathways (Notch, AKT, NF-kB) in ALDH1^+^ from breast cancer, label-retaining cells in bladder cancer, CD133^+^CD44^+^ cells in CRC and sphere-derived cells from ovarian cancer (Hirata et al., 2015; Kurtova et al., 2015; Wang et al., 2015; Seo et al., 2016).

### Alternative Fuels

Cancer cells require the use of amino acids for their heightened metabolic needs. Indeed, one of the most important metabolic pathways for cancer cells is that related to glutamine (Wise and Thompson, 2010), since it is an important substrate for DNA and fatty acid synthesis, as well as anaplerosis of the TCA cycle. Indeed, glutamine addiction has become a hallmark of glycolytic tumors, especially those with increased c-MYC expression (Deberardinis and Cheng, 2010; Wise and Thompson, 2010; Korangath et al., 2015). In addition, glutamine is related to glutathione synthesis, well known for its powerful antioxidant ability and some other biological activities (Todorova et al., 2004; Son et al., 2013). Although OxPhos-dependent pancreatic CD133^+^ CSCs are resistant to glutamine deprivation (Sancho et al., 2015), evidence of the involvement of glutamine metabolism in the maintenance of the stem-like SP phenotype has been provided in lung and pancreatic cancer by a β-catenin/redox-mediated mechanism (Liao et al., 2017). In fact, glutamine deprivation in pancreatic cancer cell lines inhibited their self-renewal capacity, reduced their stemness gene signature and increased sensitivity to radiotherapy (Li D. et al., 2015). Additionally, aminoacid metabolism, especially glutamine, is increased in acute myeloid leukemia (AML) ROS^low^ CSCs to fuel OxPhos and favor survival (Jones et al., 2018). Interestingly, leukemia CSCs may obtain their glutamine supply from neighbor stromal cells, as described for bone marrow adipocytes supporting cancer cells growth after asparaginase treatment in high-risk leukemia patients (Ehsanipour et al., 2013).

Apart from glutamine, the metabolism of amino acids, such as lysine or serine may also support CSCs features. Indeed, CRC CD110^+^ tumor-initiating cells (TICs) are rich in enzymes implicated in both lysine transport and catabolism, which activates β-catenin-dependent Wnt signaling, ultimately promoting self-renewal and metastasis (Wu Z. et al., 2015). Additionally, accumulation of alpha-aminoadipate, an intermediate of lysine catabolism, on brain TICs correlates with poor survival rate of glioblastoma patients, representing a marker of tumor aggressiveness (Rosi et al., 2015). On the other hand, recent data demonstrate that phosphoglycerate dehydrogenase (PHGDH), catalyzing the first step of the serine biosynthesis, maintains self-renewal and tumorigenicity of lung, breast and brain CD133^high^ sphere-forming cells in a mechanism involving pluripotency gene expression and redox balance (Samanta et al., 2016; Sharif et al., 2018). Finally, endogenous tryptophan derivatives, such as Kyn (kynurenine) and ITE (2-(10H-indole-30- carbonyl)-thiazole-4-carboxylic acid methyl ester), may play opposite roles on cancer progression and stemness, regulating OCT4 expression through aryl hydrocarbon receptor (AhR) modulation: accumulation of the low-affinity AhR agonist Kyn in the tumor microenvironment favor carcinogenesis, whereas the high-affinity AhR agonist ITE promotes its binding to the OCT4 promoter to suppress its transcription and, consequently, inducing cell differentiation in U87 glioblastoma neurospheres (Cheng et al., 2015).

Ketone bodies can also work as fuel to promote tumor growth and play a role in CSCs activity. Reports on breast cancer showed the role of ketone bodies increasing the expression of stemness-related genes, driving recurrence and metastasis, thus related to decreased patient survival (Bonuccelli et al., 2010; Martinez-Outschoorn and Lisanti, 2014).

The PPP has also come up as an alternative way to generate energy in CSCs. For instance, glioblastoma stem-like cells are remarkably metabolically flexibles, switching their metabolism depending on oxygen levels fluctuations: from high levels of PPP activity linked to active proliferation under acute oxygenation, to a glucose-dependent phenotype under hypoxia, when cell migration is stimulated (Kathagen et al., 2013). Additionally, Debeb et al. (2016) described that PPP inhibitors reduced the stemness-related markers in node-positive invasive breast carcinoma and a high rate in PPP activity was also reported in combination with an OxPhos-dependent phenotype in CD44^+^CD117^+^ CSCs from epithelial ovarian cancer (Pasto et al., 2014).

Overproduction of hyaluronan, a component of the extracellular microenvironment, supports self-renewal in human head and neck squamous cell carcinoma HSC-3 cells (Bourguignon et al., 2012) and dedifferentiation in breast cancer cells (Chanmee et al., 2014). Using metabolomic approaches, Chanmee et al. (2016) later described that increased hyaluronan production leads to a HIF1α-induced metabolic reprogramming toward glycolysis, thus creating a positive feedback loop through the hexosamine biosynthetic pathway (HBP). Interestingly, HBP inhibition considerably reduced the content of CD44^high^CD24^low^ cells and mammosphere-forming capacity.

Finally, purine metabolism has also been described to regulate stemness-related properties. Indeed, upregulation of MYC-mediated *de novo* purine synthesis maintained self-renewal, proliferation and tumor forming capacity in brain TICs, and was associated with poor prognosis in glioblastoma patients (Wang et al., 2017c).

### Considerations on the Metabolic Heterogeneity of CSCs

As inferred from the information above, CSCs display a plethora of metabolic phenotypes diverging from the classical OxPhos/Warburg phenotypes (Table 3). However, such diversity cannot be completely attributed to the intrinsic heterogeneity of cancer, since conflicting data can be often found in the literature even for the same tumor entity.

The main source of reported disparities is the utilized model systems. On the one hand, the term CSC tend to be loosely used and include models as dissimilar as established cell lines grown as spheroids in 3D and sorted cells from human tumors expressing one or several surface markers. In fact, although resistance to anoikis and the ability to grow in anchorage-independent conditions are well-accepted characteristics of stem-like cells, the percentage of *bona-fide* CSCs within a spheroid may be as low as 1%. On the other hand, established cell lines are usually clonal and have been passaged *in vitro* for dozens or even hundreds of times: resemblance with the genetic and phenotypic heterogeneity found in tumors barely exists. Even when considering sorted cells from fresh tumors, we need to bear in mind that most surface markers are not completely reliable and may be lost or modified in sample preparation: in fact, trypsinization time may greatly affect expression of these markers. Most importantly, most *in vitro* studies are carried out in artificial metabolic conditions (e.g., high glucose and oxygen) lacking microenvironmental components of the CSC niche. In fact, tumor niche can support metabolic alterations in CSCs (Mateo et al., 2014; Ye et al., 2016) via signaling and metabolic crosstalk.

Interestingly, even with these limitations, different groups have found phenotypically diverse CSCs subpopulations coexisting in the same *in vitro* or *in vivo* conditions (Diehn et al., 2009; Sancho et al., 2015; Luo et al., 2018). For instance, although most pancreatic CSCs are dependent on OxPhos, a pre-existing subpopulation of CD133^+^ resistant to mitochondrial inhibition, due to their increased metabolic plasticity, was detected (Sancho et al., 2015). Importantly, differences in metabolism may be associated to functional diversity inside the CSCs population. Indeed, metabolic heterogeneity, mainly in terms of redox state, has been associated to differences in stemness, as well as chemo and radioresistance (Diehn et al., 2009; Wang et al., 2013; Sancho et al., 2015; Ye et al., 2016). Additionally, several reports link CSCs with enhanced metastatic abilities to specific metabolic traits. Indeed, reduced mitochondrial DNA and function contribute to the acquisition of a metastatic phenotype in spheroid-forming breast cancer cells (Guha et al., 2014). Moreover, alterations in redox balance leading to NRF2 activation mediate a phenotypic switch from glycolytic mesenchymal-like to OxPhos-dependent epithelial-like breast CSCs (Luo et al., 2018). In addition to breast cancer, CD44^+^ESA^low^ cells with increased metastatic potential (upon EMT), linked to low ROS levels, as compared to their non-EMT counterparts, have been described for oral and skin carcinomas, as well as prostate cancer (Gammon et al., 2013; Aguilar et al., 2016). On the contrary, metastasis-initiating cells in melanoma bear redox stress, as inferred from their elevated ROS and reduced glutathione content (Porporato et al., 2014; Piskounova et al., 2015).

## Therapeutic Targeting of CSCs Metabolism

Considering the involvement of CSCs in chemoresistance, tumor relapse and metastasis, there is a pressing need in cancer therapy to find new strategies to eradicate this aggressive cell population. As summarized in the previous section, the distinct metabolic features of CSCs, compared to non-CSCs, constitute a significant opportunity to targeting specifically the CSCs component of tumors and eradicate the tumor bulk.

### Mitochondrial Metabolism

**FIGURE 1:**
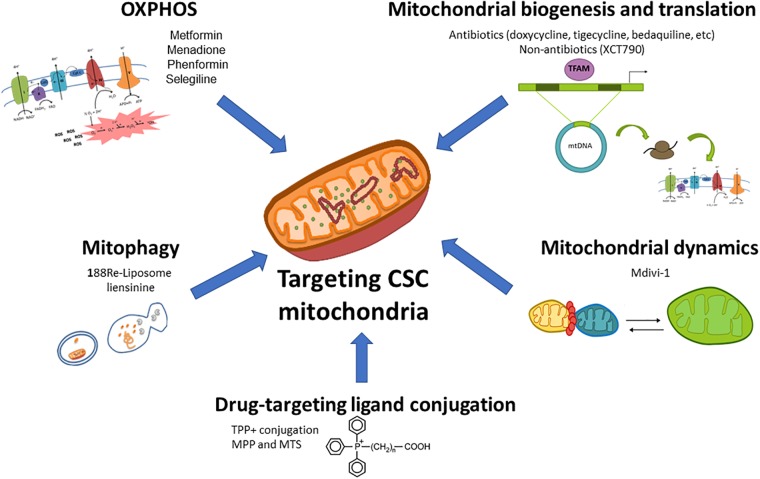
Therapeutic targeting of mitochondrial metabolism in CSCs. Different aspects of the mitochondrial metabolism can be approached to target CSCs: (1) oxidative phosphorylation (OxPhos) can be impaired by ETC inhibitors such as the antidiabetic drugs metformin or phenformin, the reactive oxygen species (ROS) inductor and complex I inhibitor menadione, or the anti-Parkinson compound selegiline; (2) Mitochondrial biogenesis and translation can be targeted by FDA-approved antibiotics such as doxycycline, tigecycline, bedaquiline among others, or non-antibiotic inhibitors; (3) Mitochondrial dynamics can be disrupted by the mitochondrial division inhibitor Mdivi-1; (4) The blockage of mitophagy, an essential mitochondrial quality control system, with nanomedicines such as 188Re-liposome or the inhibitor liensinine may affect CSCs functions; (5) The use of nanocarriers (lipophilic cations, peptides and nanoparticles) conjugated with chemotherapeutics and small drugs may be used for a selective delivery of drugs in mitochondria.

As mentioned above, mitochondria play a key role for CSCs functionality regardless of their dominant metabolic phenotype, suggesting that targeting mitochondrial metabolism may be the most effective therapeutic strategy for their elimination. Noteworthy, highly glycolytic cells with mutations in the TCA cycle or ETC still require functional mitochondria for the generation of metabolites from glutamine via reductive carboxylation (Mullen et al., 2011). For those reasons, pharmacological approaches designed to target different aspects of mitochondrial function for cancer treatment are currently under intense investigation in preclinical and clinical studies (Figure 1).

#### Targeting OxPhos

Inhibition of mitochondrial respiration by compounds blocking ETC complexes is one of the most studied metabolism-based strategies for cancer treatment. In fact, tumor cells in nutrient-deprived environments or displaying limited metabolic plasticity, as described for some gliomaspheres and PDAC CD133^+^ cells (Janiszewska et al., 2012; Sancho et al., 2015), have restricted ability to cope with decreased mitochondrial ATP production. ETC inhibitors also target glycolytic CSCs, such as CD44^+^CD24^low^ cells in breast cancer or SP cells in nasopharyngeal carcinoma (Vazquez-Martin et al., 2010; Shen et al., 2013), highlighting the importance of ETC for coupled ATP production, avoiding electron loss in the form of ROS.

One of the most studied ETC inhibitors in the context of CSCs targeting is the antidiabetic drug metformin. Reported antitumoral effects of this drug relate to both systemic glucose decrease, and direct cancer cell targeting via ETC complex I inhibition (Wheaton et al., 2014). Although metformin shows cytostatic properties at low concentration, it induces apoptosis specifically in PDAC CD133^+^ cells and CD44^high^ CD24^low^ mammospheres (Iliopoulos et al., 2011; Sancho et al., 2015), which, at least for PDAC CD133^+^ cells, is attributable to their dependency on mitochondrial metabolism. Although metformin clinical testing for pancreatic cancer treatment showed no improvement in patient survival rate (Kordes et al., 2015; Reni et al., 2016), positive clinical data has been reported for breast, endometrial and prostate cancer. On the other hand, the development of resistance to metformin monotherapy *in vivo* suggests that the design of combinatory treatments (Sancho et al., 2015; Roh et al., 2017) or the use of stronger mitochondrial inhibitors may be needed. This could be the case of the ETC complex I inhibitor phenformin, which is more efficiently delivered to mitochondria (Petrachi et al., 2017) and when combined with the ALDH inhibitor gossypol, suppresses stemness, invasiveness and cell viability in glioblastoma (Park et al., 2018). Additionally, menadione, with dual mechanism of action combining complex I inhibition and ROS induction, prevents the development of resistance (Sancho et al., 2015).

Following metformin’s relative success, a great effort has been put on the drug repurposing for CSCs targeting in cancer treatment. In this sense, several known FDA-approved antibiotics target the ETC at different levels and have proven to selectively decrease CSC content. This is the case of antimycin A, a powerful complex III inhibitor that decreased lung spheroids (Yeh et al., 2013); the antituberculosis agent bedaquiline (complex V inhibitor) that targeted mammospheres (Fiorillo et al., 2016); oligomycin (another complex V inhibitor), which showed drastic synergistic effects suppressing cell growth and motility in glioblastoma cell lines when combined with 2-deoxy-D-glucose (2DG) (Kennedy et al., 2013); and niclosamide, an antihelmintic with ETC uncoupling properties, that inhibited TICs from ovarian and breast cancers (Yo et al., 2012; Wang et al., 2013). Similarly, numerous studies suggest the efficacy of the OxPhos inhibitor salinomycin for CSCs targeting *in vitro* and *in vivo* in diverse cancer types (Naujokat and Steinhart, 2012 and references therein). In this last case, however, the final antitumoral effect may be the result of a combination of factors, since salinomycin also interferes with ABC transporters or Wnt signaling (Naujokat and Steinhart, 2012). Besides antibiotics, the agent L-deprenyl (also known as Selegiline), a monoamine oxidase-B (MAO-B) inhibitor typically used for the treatment of Parkinson’s disease, was found to exert antimitochondrial activity and cause apoptotic cell death in AML CSCs through the reduction of ETC and glycolysis-related gene expression, independently of MAO-B inhibition (Ryu et al., 2018).

Considering the therapeutic potential of OxPhos inhibition, compound discovery is currently taking place in order to identify new selective molecules with adequate *in vivo* properties. As an example, the compound VLX600 showed cytotoxicity in colon cancer spheroid-derived cells both *in vivo* and *in vitro*, by directly inhibiting ETC complexes in metabolically compromised microenvironments (Zhang et al., 2014).

#### Targeting Mitochondrial Translation and Biogenesis

As commented above, several FDA-approved antibiotics can disrupt mitochondrial function. Apart from direct OxPhos inhibition, certain widely prescribed antibiotics target either mitochondrial translation or biogenesis as an “off-target” effect (Lamb et al., 2015c), inhibiting self-renewal ability of multiple tumor types (Lamb et al., 2015a,b). For instance, the use of a tetracycline such as doxycycline induced apoptosis in pancreatic cancer cell lines and human cervical carcinoma tumorspheres (Son et al., 2009; Yang et al., 2015), while azithromycin (erythromycin family) demonstrated to inhibit the self-renewal capacity of PDAC spheroids (Lamb et al., 2015c). On the other hand, the use of the antimicrobial tigecycline selectively killed leukemia CD34^+^CD38^-^ cells without affecting normal hematopoietic cells through the inhibition of mitochondrial translation (Skrtic et al., 2011).

However, although this research line shows promising results, continuous treatment with antibiotics for cancer therapy may be ineffective (Esner et al., 2017). Indeed, long-term desensitization was reported for human metastatic breast cancer cells treated with different antibiotic classes including streptomycin, tetracycline, kanamycin, G418-geneticin (aminoglycoside), puromycine (aminonucleoside) and blasticidine (Esner et al., 2017). Nonetheless, the design of novel combinatory treatments or stronger derivatives could overcome this setback for future application in the clinical setting, taking advantage of the well-known safety profile of antibiotics.

On the other hand, non-antibiotic inhibitors of mitochondrial biogenesis are already available: XCT790, a specific inhibitor of the estrogen-related receptor alpha (ERRα)/PGC-1α, signaling pathway (responsible for mitochondrial biogenesis), inhibited breast CD44^+/high^CD24^-/low^ TICs and mammosphere survival and propagation by reducing OxPhos. These effects were prevented or reversed by stimulating mitochondrial biogenesis with the mitochondrial fuel acetyl-L-carnitine (ALCAR9) (De Luca et al., 2015).

#### Targeting Mitochondrial Dynamics

Several types of cancer show downregulation of mitochondrial fusion proteins (Zhang et al., 2013; Chen and Chan, 2017) or upregulation of fission proteins (Rehman et al., 2012; Kashatus et al., 2015; Wieder et al., 2015; Zhang et al., 2017). In fact, increased mitochondrial fragmentation has also been involved in malignancy, promoting tumor migration and invasion in breast cancer (Zhao et al., 2013). On the other hand, mitochondrial dynamics seem to regulate proliferation and survival of CSCs, similar to what is known in embryonic stem cells (Chen and Chan, 2017). Indeed, dynamin-related protein 1 (DRP1)-dependent fission regulates mitochondrial distribution in asymmetrical division, ensuring maximal mitochondrial fitness in the daughter stem cell (Katajisto et al., 2015).

Currently, the only available pharmacological strategy to target mitochondrial dynamics is the DRP1 inhibitor mdivi-1. On the one hand, mdivi-1 or DRP-1 knockdown reduced proliferation and induced apoptosis in lung cancer cells, whose mitochondria were in a situation of constant fission *in vitro* and *in vivo* (Rehman et al., 2012). This inhibitor also attenuates lung cancer and mesothelioma proliferation when combined with the MET inhibitor MGCD516 (Wang J. et al., 2018). Importantly, mdivi-1 reduced tumorsphere-forming ability of breast, lung, and melanoma cancer cell lines (Peiris-Pagès et al., 2018) and reduced tumorigenicity of brain TICs *in vitro* and *in vivo* (Xie et al., 2015).

#### Targeting Mitophagy

Mitophagy is an essential quality control system to selectively remove damaged, non-functional or unnecessary mitochondria in cells. However, its involvement in cancer is controversial, since contradictory reports on the role of this process in tumorigenesis have been published (Chourasia et al., 2015a).

On the one hand, the mitophagy promoter Parkin is frequently deleted in many cancer types (Cesari et al., 2003). In addition, defective mitophagy caused by BNip3 loss/inhibition promote invasion and metastasis in breast, pancreatic or CRC (Okami et al., 2004; Chourasia et al., 2015b; Li et al., 2018). Moreover, the induction of mitophagy and mitoptosis by salinomycin treatment led to decreased mitochondrial mass and ATP depletion in prostate cancer and breast cancer CD44^high^CD24^low^ cells triggering a cytotoxic effect specific to tumor cells without damaging normal fibroblasts (Jangamreddy et al., 2013).

On the other hand, mitophagy can be triggered as a stress response against nutrient deprivation or hypoxia, promoting cell survival and tumorigenesis in hostile environments and contributing to drug resistance in human CRC CD133^+^CD44^+^ cells (Jangamreddy et al., 2013; Yan et al., 2017). In agreement with this notion, mitophagy is upregulated in esophageal squamous cell carcinoma undergoing EMT (Whelan et al., 2017). Accordingly, the use of mitophagy blockers, such as the nanomedicine 188Re-Liposome or the inhibitor liensinine, reversed drug resistance in ovarian cancer cells *in vitro* (Chang et al., 2017) and in breast cancer xenografts *in vivo* (Zhou et al., 2015), respectively. Additionally, the alkaloid matrine induced mitochondrial dysfunction and apoptosis by inhibiting mitophagy in HepG2 hepatoblastoma cells (Wei et al., 2018). Interestingly, a link between autophagy and mitochondrial respiration has recently been reported: the novel autophagy inhibitor aumitin blocks complex I activity (Robke et al., 2018), while pharmacologic or genetic inhibition of complex I inhibitor impairs autophagy (Thomas et al., 2018).

#### Mitochondrial Drug Delivery

In order to ensure an efficient and selective delivery in mitochondria, different small compounds or chemotherapeutic drugs can be conjugated with nanocarriers, including lipophilic cations, peptides and nanoparticles, with preferential accumulation in mitochondria.

One of the most studied strategies involves the conjugation of small compounds with delocalized lipophilic cations, such as triphenylphosphonium (TPP), dequalinium or rhodamine 123, that possess both lipophilicity and a positive charge, and accumulate in the mitochondrial matrix (Murphy, 2008). Importantly, as OxPhos-dependent CSCs show an elevated mitochondrial membrane potential (ΔΨm), indicative of increased activity (Sancho et al., 2015), conjugated compounds will be delivered primarily to these cells. For example, MitoChromanol (vitamin E analog) or Gamitrinib (chaperone inhibitor) combined with TPP inhibit OxPhos and ATP production selectively in cancer cells (Chae et al., 2012; Cheng et al., 2013).

Commonly used chemotherapeutic agents can also be joined with lipophilic cations to exert their therapeutic action in the mitochondria and improve their effect. For example, doxorubicin (DOX) combined with TPP showed enhanced toxicity against DOX-resistant MDA-MB-453 breast cancer cells, even though TPP-DOX was as toxic as free DOX in wild type cells (Han et al., 2014). Interestingly, DOX fused with TPP-conjugated chitosan nanoparticles exhibited higher cytotoxicity than free doxorubicin in A549 and Hela cells (Hou et al., 2017). Additionally, the development of a cisplatin prodrug combined with TPP showed promising results treating cisplatin-resistant, aggressive cancers, such as neuroblastoma, since its delivery into the mitochondrial matrix circumvents the nucleotide excision repair pathway present in the nucleus (Marrache et al., 2014). Moreover, the union of paclitaxel with TPP also resulted in enhanced antitumor effects in Hela and in mouse mammary carcinoma cells (4T1) *in vitro* and *in vivo* (Biswas et al., 2012).

An alternative delivery strategy tested for chemotherapeutic agents involved their conjugation with mitochondria-penetrating peptide (MPP) or mitochondria-targeting sequences (MTS), which act independently of mitochondrial potential. This approach directs their activity toward mtDNA, thus promoting drug selectivity for cancer cells with reduced mtDNA integrity, while their stable mitochondrial location prevents the acquisition of resistance due to drug efflux (Chamberlain et al., 2013). As an example, cisplatin linked to MMP overcomes tolerance in cisplatin-resistant ovarian cancer 2780/CP70 cells (Marrache et al., 2014).

Finally, the combination of the antibiotic salinomycin with reduced graphene oxide-silver nanocomposites synergistically enhanced the activity of either compound alone, leading to mitochondrial dysfunction and selectively killing human ovarian CD133^+^ cells (Choi et al., 2018).

### Targeting Redox Homeostasis

It is well established that intracellular ROS accumulation induces cancer cell death, a strategy widely used in the clinics associated to classical chemo and radiotherapy. However, recent evidence suggests that this approach may not be effective against CSCs, due to their increased antioxidant potential (Diehn et al., 2009; Ishimoto et al., 2011; Yuan et al., 2015). Moreover, ROS can be a double-edged sword, since they may promote CSCs survival and invasive abilities acting as signaling molecules (Luo et al., 2018).

As previously mentioned, CSCs are characterized by a finely regulated redox metabolism (Le Belle et al., 2011; Paul et al., 2014; Chang et al., 2018), where glutathione plays an essential role to maintain stemness characteristics (Diehn et al., 2009; Ishimoto et al., 2011). For that reason, increasing oxidative stress by blocking glutathione synthesis could represent a novel therapeutic strategy for eliminating CSCs population and diminishing tumor growth (Diehn et al., 2009; Rodman et al., 2016). Thus far, buthionine sulfoximine (BSO), an inhibitor of glutathione biosynthesis, has proven (Marengo et al., 2008) to be very effective in decreasing clonogenicity and enhancing response of CSCs to radiotherapy *in vitro* and *in vivo* (Diehn et al., 2009; Boivin et al., 2011; Rodman et al., 2016). Due to its importance for glutathione biosynthesis, especially in glutamine-addicted cancer cells, deprivation of glutamine increased oxidative stress and reduced SP cells in non-small lung and pancreatic cancer cell lines (Liao et al., 2017). Glutamine deprivation also inhibited metastatic potential of cancer cells, one of the main characteristics of CSCs (Wang et al., 2010).

**FIGURE 2:**
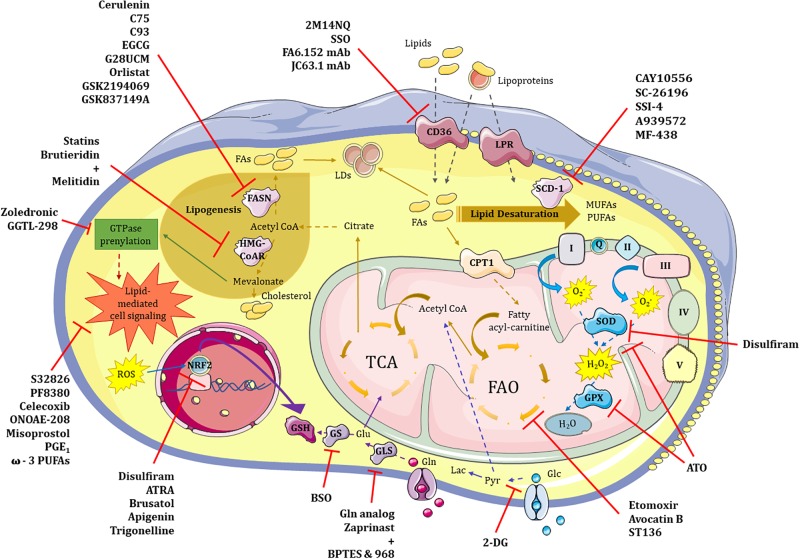
Therapeutic targeting of glycolysis, lipid and redox metabolism in CSCs. Metabolic pathways such those involving glucose, lipids and redox balance are potentially targetable in CSCs. (1) *Glycolysis*. 2-DG represent the most promising therapeutic approach to neutralize highly glycolytic CSCs in combination treatments. (2) *Lipid metabolism*. 2M14NQ, SSO and the monoclonal antibodies FA6.152 and JC63.1 can block CD36 activity; substances like Etomoxir, Avocatin B or ST136 block fatty acid oxidation (FAO) in the mitochondria; FASN can be inhibited by drugs such as Cerulenin, C75, C93, EGCG, G28UCM, Orlistat, GSK2194069 or GSK837149A; while HMG-CoAR enzyme may be inhibited by either Statins or the combination of Brutieridin plus Melitidin; GTPase prenylation pathway in which mevalonate is involved can be targeted by both Zoledronic acid and GGTL-298; and different steps of the lipid-mediated cell signaling may be blocked with molecules such as S32826, PF8380, Celecoxib, ONOAE-208, Misoprostol, PGE_1_ and ω-3 PUFAs; finally, targeting of the main enzyme of lipid desaturation route, SCD-1, can be achieved by CAY10556, SC-26196, SSI-4, A939572 or MF-438. (3) *Redox metabolism*. Antioxidant features of CSCs may be inhibited at different levels including SOD and GPX proteins with Disulfiram and/or ATO, respectively; ROS-induced NRF2 activity can be neutralized by Disulfiram, ATRA, Brusatol, Apigenin and Trigonelline; finally, glutathione synthesis may be inhibited either directly or indirectly by blocking GS or GLS enzymes with BSO or a glutamine analog, and a mixture of Zaprinast with BPTES or 968 compounds, respectively. 2-DG – 2-deoxy-D-glucose, Pyr – pyruvate, LDs – lipid droplets, LPR – lipoprotein receptor, FAs – fatty acids, FASN – fatty acid synthetase, HMG-CoAR – 3-hydroxy-3-methyl-glutaryl-coenzyme A reductase, SCD-1 – stearoyl-CoA desaturase, MUFAs – monounsaturated fatty acids, PUFAs – polyunsaturated fatty acids, FAO – fatty acid oxidation, TCA – tricarbolxylic acid cycle, CPT1 – carnitine palmitoyltransferase I, GTPase – guanosin triphosphatase, I/Q/II/III/IV/V – complexes of the electron transport chain, O_2_^-^ – superoxide anion, H_2_O_2_ – oxygen peroxide, SOD – superoxide dismutase, GPX – glutathione peroxidase, ROS – reactive oxygen species, NRF2 – nuclear factor erythroid 2–related factor 2, GSH – glutathione, Glu – glutamate, Gln – glutamine, GS – glutathione synthase, GLS – glutaminase, 2M14NQ – 2-methylthio-1,4-naphtoquinone, SSO – sulfosuccinimidyl oleate, mAb – monoclonal antibody, EGCG – epigallocatechin gallate, ATRA – all-trans retinoic acid, BSO – L-buthionine-S,R-sulfoximine, ATO – arsenic trioxide.

Besides glutathione, strategies aimed at inhibiting cellular antioxidants are currently applied with relative success, mostly improving response to conventional therapies (Figure 2). For example, treatment with auranofin, a thioredoxin reductase inhibitor, increased the sensitivity of human breast CSCs to radiotherapy (Rodman et al., 2016), while a synergistic reduction of the CD44v9^+^ cells content was achieved by inhibiting glutathione-S-transferase and thioredoxin reductase in patient-derived xenograft (PDX) models of CRC (Tanaka et al., 2016). Arsenic trioxide (ATO), an FDA-approved drug for acute promyelocytic leukemia that increases ROS content and depletes superoxide dismutase (SOD) and glutathione peroxidase (GPX) (Li et al., 2006), proved to reduce CSCs content in different cancer types (Ding et al., 2014; Li et al., 2015a; Chang et al., 2016; Bell et al., 2018). Moreover, the synergistic effect of ATO with glutathione depletion could present a novel treatment for cancers unresponsive to ATO treatment alone (Miller, 2002; Davison et al., 2003; Bhalla et al., 2009; Matulis et al., 2012). The anti-alcohol addiction drug disulfiram has been widely used as anticancer agent since it can increase oxidative stress by blocking SOD activation (Calderon-Aparicio et al., 2015) and inhibiting NRF2 (Xu et al., 2017). In studies with breast cancer cell lines *in vitro* and *in vivo*, disulfiram not only diminished mammosphere formation (Yip et al., 2011; Kim et al., 2017) and reduced CD44^+^CD24^-^ and CD49f^+^CD24^+^ subpopulations, but also managed to reverse paclitaxel and cisplatin resistance of triple-negative breast cancer (TNBC) (Liu et al., 2013). Moreover, disulfiram proved to diminish the ALDH1^+^ population from non-small cell lung cancer cell lines (Liu et al., 2016) and CD34^+^CD38^+^ cells in AML cell lines and primary samples *in vitro* and *in vivo* (Xu et al., 2017). Now entering phase III in clinical trials, disulfiram may present a potential adjuvant therapy for cancer treatment, although it is highly unstable in blood. For that reason, disulfiram-containing nanoparticles have also been developed. Even though these nanoparticles proved to increase disulfiram blood levels (Song et al., 2016), further studies are needed to establish its full *in vivo* antioxidant and biological properties.

Increased NRF2 levels turned out to play a role in CSCs survival and chemoresistance (Kwak et al., 2001; Zhu et al., 2013; Ryoo et al., 2016), representing another potential target for eradicating CSCs. All-trans retinoic acid (ATRA) blocked NRF2 activation, diminishing self-renewal and tumorigenic capacity of ALDH1^+^ lung cancer cells (Moreb et al., 2004) and ovarian cancer cell lines (Kim D. et al., 2018). Moreover, brusatol, which decreases NRF2 protein levels, was demonstrated to inhibit mammospheres formation and increase the sensitivity of human breast CSCs to Taxol (Wu T. et al., 2015). Similarly, the natural flavonoid apigenin (Kim et al., 2016; Erdogan et al., 2017) or the alkaloid trigonelline (Arlt et al., 2013; Roh et al., 2017), which inhibits NRF2 at transcriptional and translational level, can sensitize CSCs toward chemotherapeutic drugs. On the other hand, the combination of ROS inducers and NRF2 (or downstream targets) inhibition could represent a potential strategy for CSCs elimination (Diehn et al., 2009; Ishimoto et al., 2011; Kwak et al., 2016; Liu et al., 2016; Kim et al., 2017; Kim D. et al., 2018; Luo et al., 2018).

Paradoxically, some natural antioxidants that can increase NRF2 expression levels have also shown therapeutic potential. The NRF2-inducer sulforaphane, a dietary component from broccoli, inhibited self-renewal capacity of CD44^+^ LDH1^+^ pancreatic (Rausch et al., 2010) and ALDH1^+^ breast cancer cells *in vivo* and *in vitro* (Burnett et al., 2017). Curcumin, an active ingredient of turmeric, diminished self-renewal capacity of CD44^+^ EpCAM^+^ pancreatic cancer cells *in vitro* and *in vivo* (Bao et al., 2012) and reduced proliferation and mammosphere formation of ALDH1^+^ breast cancer cells (Kakarala et al., 2010). Additionally, resveratrol, oleanane triterpenoid or carnosol also proved to active and increase NRF2 expression which could have a positive effect on diminishing the CSCs population (Probst et al., 2015a,b; Sancho et al., 2015; Giacomelli et al., 2017). Treatment with naturally occurring antioxidants, such as vitamin C or phenethyl isothiocyanates (PEITCs), found in broccoli or Brussel sprouts, also diminished self-renewal capacity and clonogenicity of NCCIT human embryonic carcinoma and human colon cancer cell lines, and reduced CD133^+^, EpCAM^+^ and OV6^+^ cells while inhibiting tumorspheres formation and growth of hepatocellular carcinoma cell lines and PDX models *in vivo* (Yun et al., 2017; Lv et al., 2018). However, even though natural antioxidants could represent an exciting strategy in anticancer therapy, clinical trials thus far showed no positive effect on patient survival. Indeed, published data highlighted the lack of specificity of antioxidant treatments. This fact, together with the possible contribution of antioxidants to stemness maintenance and cancer development, weakens the translation potential of this approach.

### Targeting Lipid Metabolism

Lipid metabolism has become an interesting target in order to design new anti-CSC strategies, and a number of compounds have been tested during the last years (Figure 2).

#### Lipid Desaturation

Over the last few years, several SCD-1 inhibitors have demonstrated their effectiveness in different preclinical *in vitro* and *in vivo* models of cancer, by specifically targeting stemness-related properties. Indeed, the inhibitors CAY10556 and SC-26196 reduced stem cell-related markers and signaling pathways by downregulating Hedgehog and Notch expression in ovarian ALDH^+^CD133^+^ cells (Li J. et al., 2017). Interestingly, this led to the inhibition of sphere formation *in vitro* and tumorigenicity *in vivo*, with no effect on differentiated cells, suggesting the selectivity of this approach. In the same line of evidence, inhibition of SCD-1 with the compounds SSI-4 or A939572 modulates endoplasmic reticulum-stress-mediated differentiation in liver chemoresistant hepatospheres, sensitizing resistant PDXs to sorafenib treatment with low side toxicity *in vivo* (Ma et al., 2017). In parallel, effects of the inhibitor A939572 in CD133^+^CD49f^+^ liver CSCs have also been linked to Wnt-mediated self-renewal and *in vivo* tumorigenicity (Lai et al., 2017). Finally, MF-438 treatment induced anoikis in lung ALDH1^+^ cells, decreasing self-renewal and pluripotency markers expression (Pisanu et al., 2017). Interestingly, these *in vitro* effects translated into reduced tumorigenic potential and reversion of chemoresistance *in vivo*.

#### Lipogenesis

Given the important involvement of the enzyme FASN in numerous tumor types, a number of inhibitors have been designed and/or tested in diverse cancer models: cerulenin, C75, C93, epigallocatechin gallate (EGCG), G28UCM, orlistat, GSK2194069 and GSK837149A. In fact, cerulenin treatment prevents proliferation *in vitro* of pancreatic spheres (Brandi et al., 2017) and neurospheres established from glioma patients (Yasumoto et al., 2016) CSCs. On the other hand, C75 at non-cytotoxic concentrations significantly reduced self-renewal in HER2^+^ breast cancer cells (Corominas-Faja et al., 2017). However, it is important to highlight the critical selectivity and toxicity issues found for FASN inhibitors *in vivo*, which have compromised their translation to clinical trials. Only the inhibitor TVB-2640 is being currently tested in clinical trials for HER2^+^ advanced breast cancer, high grade astrocytoma and colon cancer (NCT03179904, NCT03032484, NCT02980029, respectively).

#### Cholesterol Synthesis

Cholesterol synthesis through the mevalonate pathway can be inhibited by statins, for which the molecular target is the enzyme 3-hydroxy-3-methylglutharyl-coenzyme A reductase (HMG-COAR). In fact, treatment with different statins decreased self-renewal and CSCs content in breast (Ginestier et al., 2012) and nasopharyngeal (Peng et al., 2017) carcinomas. Interestingly, similar effects were detected in CD133^+^ brain TICs (Wang et al., 2017a) where overexpression of mevalonate pathway genes was controlled by MYC, highlighting the variety of metabolic pathways controlled by the oncogene. However, anti-CSCs effects of statins could also be related to inhibition of cellular signaling via small GTPases (e.g., Rho and Rac), since they require prenylation using mevalonate pathway intermediates. In fact, impaired self-renewal ability achieved with simvastatin treatment in breast tumorspheres was recapitulated by zoledronic acid and GGTI-298, inhibitors of the prenylation pathway (Ginestier et al., 2012). Moreover, a mixture of brutieridin and melitidin (natural products derived from bergamot with statin-like properties) impaired breast ALDH1^+^ CSCs proliferation inhibiting both FAO and Rho-related signaling pathways (Fiorillo et al., 2018).

#### Lipid Uptake

Strategies targeting lipid uptake are mainly designed to inhibit the transporter CD36, by either pharmacological inhibition or blocking antibodies. CD36 blockade with 2-methylthio-1,4-naphtoquinone decreases self-renewal ability and induces apoptosis in glioblastoma CD133^+^ (Hale et al., 2014). Another CD36 inhibitory compound, sulfosuccinimidyl oleate, decreases chemoresistant leukemic stem cells (Ye et al., 2016). Interestingly, CD36-neutralizing antibodies against either all known functions of CD36 (FA6.152) or the ones reported to block active FA and lipoprotein uptake (JC63.1) induced lipotoxicity in label-retaining/CD44^+^ metastasis-initiating cells, thus, inhibiting metastasis initiation and progression in oral squamous cell carcinoma, with no reported toxicity *in vivo* (Pascual et al., 2016).

#### FAO

Fatty acid oxidation inhibition with etomoxir has been studied in preclinical *in vitro* and *in vivo* cancer models. Indeed, etomoxir treatment inhibits mammosphere formation and tumor growth *in vivo* in TNBC tumors bearing high MYC expression (Camarda et al., 2016). In addition, etomoxir treatment sensitizes hepatocarcinoma CD133^+^CD49f^+^ CSCs to standard chemotherapy with sorafenib (Chen C.L. et al., 2016). Moreover, etomoxir decreases the number of quiescent leukemia CSCs in AML patients and, combined with the BCl-2 inhibitor ABT-737, substantially decreases tumor burden (Samudio et al., 2010). However, etomoxir treatment induces normal hematopoietic stem cell exhaustion invalidating this compound for further clinical studies (Ito et al., 2012). Interestingly, alternative FAO inhibitors with higher selectivity for malignant cells are under investigation currently. For example, avocatin B is a lipid that accumulates in mitochondria inhibiting FAO and targets AML cells and leukemia CD34^+^ CSCs with no effect on hematopoietic stem cells (Lee et al., 2014, 2015). Additionally, the compound ST136 showed antileukemic activity with no effect on normal CD34^+^ stem cells (Ricciardi et al., 2015).

#### Lipid-Mediated Signaling

As stated in the previous section, lipid-mediated signaling plays an important role in cancer and, specifically, in CSCs functions. For that reason, several therapeutic approaches, including inhibitors and indirect modulation via dietary supplements, have been studied over the last few years. For example, several stemness-related functions of ovarian spheroid-derived cells from cell lines and primary cells from patients were dependent on LPA synthesis. Thus, inhibition of the LPA-producing enzyme autotaxin with the small molecules S32826 or PF8380 decreased tumorigenicity and chemoresistance *in vivo* (Seo et al., 2016). Interestingly, inhibition of LPA production not only affects cancer cells, but could also play an important role modulating the immune system and supporting tumor progression. Indeed, LPA induces the differentiation of monocytes into macrophages and favors the activation of CAFs phenotype (Ray and Rai, 2017; Radhakrishnan et al., 2019).

The most studied lipid mediator class in relation to CSCs is prostaglandins. Indeed, treatment of Apc^Min^/þ mice with celecoxib, the prostaglandin-endoperoxide synthase 2 selective inhibitor, or the EP4 receptor (prostaglandin receptor) antagonist ONOAE-208 resulted in a reduction of tumor CD133^+^CD44^+^ cells and tumor burden (Wang et al., 2015). Importantly, celecoxib inhibited CSCs content and the number of liver metastatic tumors upon orthotopic injection of patient-derived CRC into NSG mice. Additionally, celecoxib impaired chemoresistance in bladder carcinomas, suggesting its utility as adjuvant therapy (Kurtova et al., 2015). On the contrary, activation of EP4 with the FDA-approved agonist misoprostol or PGE_1_ reduced CD34^+^ cells in a xenograft model of chronic myelogenous leukemia (CML) (Li F. et al., 2017), suggesting a context-dependent effect of prostaglandins in stemness.

On the other hand, preclinical and human observational studies suggest that dietary omega-3 polyunsaturated fatty acids (ω-3 PUFA), including eicosapentaenoic acid (EPA) and docosahexaenoic acid (DHA), decrease CRC risk and may be effective as adjuvant treatment of advanced CRC. Indeed, EPA and DHA reduced the CD133^+^ content or stem properties in two different *in vitro* studies using CRC cell lines (De Carlo et al., 2013; Yang et al., 2013). Interestingly, EPA alone or in combination with chemotherapy decreased sphere-forming ability and suppressed tumor growth, likely through inhibition of proinflammatory metabolites in mice (Vasudevan et al., 2014). Importantly, studies in other tumor entities also suggest an anti-CSCs effect of ω-3 PUFAs besides CRC. Indeed, EPA and DHA supplementation also reduced proliferation and induced toxicity in breast tumorspheres, likely through alteration of the prostaglandin profile (Erickson and Hubbard, 2010). In addition, a metabolite derived from EPA eradicated leukemia M34^+^Kit^+^Sca1^+^ CSCs in PDXs of CML (Hegde et al., 2011).

### Targeting Metabolism of Alternative Fuels

As mentioned in the previous section, CSCs may utilize a number of different substrates, such as amino acids and ketone bodies, in order to support self-renewal and tumorigenicity. For that reason, diverse compounds which target the metabolism of these alternative fuels are currently under investigation.

On the one hand, the use of a glutamine analog reduced 20 times tumor growth and inhibited metastasis in the VM-M3 murine tumor model of systemic metastasis, when compared with non-treated mice (Shelton et al., 2010). Interestingly, the anti-asthma compound Zaprinast was identified as a novel glutaminase inhibitor that, together with BPTES and 968, inhibited clonogenicity of pancreatic cancer cells in response to radiation (Elhammali et al., 2014).

Interestingly, Ozsvari et al. (2017) unveiled the potential anti-CSCs activity of a novel class of compounds denominated “mitoketoscins.” These compounds block the active site of the enzymes involved in the recycling of ketone bodies into acetyl-CoA (OXCT1 and ACAT1), leading to inhibition of the CSCs activity and propagation in breast cancer spheroids. However, considering the sometimes contradictory results of diverse studies on the antitumor effects of ketogenic diet (high-fat/low-carbohydrate intake) (Vidali et al., 2015; Weber et al., 2018), the anti-tumor effect of these inhibitors would need to be carefully tested as dependency on ketone bodies strongly varies across tumor entities and specific genotypes.

### Combination Treatments: Targeting Glycolysis

Considering the great intratumoral metabolic heterogeneity and plasticity found in tumors, mitochondrial inhibitors as single agents will unlikely become an effective therapy for cancer treatment. In fact, combination treatments, where two or more metabolic pathways are inhibited simultaneously, would block relapse and development of resistances. For instance, a dual inhibition of the main metabolic pathway together with its main escape mechanism will completely erase CSCs within the tumor. This has been reported for the combinations of metformin with either the bromodomain and extraterminal motif (BET) inhibitor JQ-1 in pancreatic cancer (Sancho et al., 2015) or PI3K inhibition for ovarian cancer (Li et al., 2012), which blocks OxPhos and indirectly inhibits glycolysis simultaneously.

In fact, direct glycolysis inhibition for cancer treatment has been studied intensively in preclinical and clinical settings over the last few years, although with low success rates. On the one hand, glucose transport inhibitors, such as silybin/silibinin [tested in a phase I/II clinical trials for prostate cancer and advanced hepatocellular carcinoma (Flaig et al., 2006)], phloretin, WZB117 and fasentin caused important side effects, since GLUT transporters are present in all the cells of the organism. Similarly, inhibition of glycolytic enzymes, such as hexokinase II with lonidamine, has been tested in several types of cancers, including breast, lung and ovarian cancer (Gadducci et al., 1994; De Lena et al., 1997; De Marinis et al., 1999; Berruti et al., 2002). However, there was no significant improvement in overall survival and many cases presented with elevated toxicity. Additionally, the glucose analog 2-DG was shown to be a promising agent in preclinical studies (Maschek et al., 2004; Coleman et al., 2008). In fact, it has been recently tested in phase II/III of a clinical trial for prostate cancer (NCT00633087), although no results are available, since the trial was terminated due to the slow accrual.

Apart from direct inhibition, targeting tumor drivers affecting cellular metabolism might hinder glycolysis. For example, KRAS mutation is present in more than 90% of pancreatic cancer cases (Bailey et al., 2016) and controls both tumorigenesis and metabolic reprogramming (Ying et al., 2012; Son et al., 2013; Liou et al., 2016). In fact, KRAS drives glycolysis and the diversion of glycolysis intermediates into the non-oxidative branch of PPP, essential for the synthesis of nucleic acids (Yun et al., 2009; Ying et al., 2012; Blum and Kloog, 2014). However, even though small molecule inhibitors of KRAS proved to be promising in preclinical studies (Xie et al., 2017; Zeng et al., 2017), targeting KRAS or its downstream pathways showed no effect in overall survival and overall response rate in pancreatic cancer patients (Kindler et al., 2012; Infante et al., 2014; Chung et al., 2017).

Alternatively, c-MYC is another essential driver of tumorigenesis and glycolysis in cancer (Miller et al., 2012; Lin et al., 2013; He et al., 2015). For that reason, different compounds targeting MYC are currently undergoing clinical trials. Noteworthy, inhibitors of BET proteins directly downregulate MYC expression and suppress tumor growth *in vivo* (Delmore et al., 2011; Mazur et al., 2015; Garcia et al., 2016). Importantly, since MYC suppression blocks development of resistance to mitochondrial inhibitors (Sancho et al., 2015; Kim J.H. et al., 2018), combinatory approaches using this strategy can represent a promising anticancer therapy.

## Concluding Remarks

**FIGURE 3:**
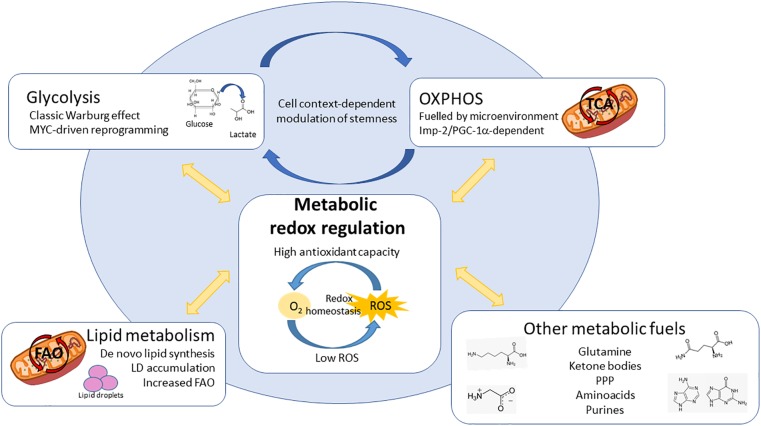
Redox involvement in the different metabolic dependencies described for CSCs. CSCs bear diverse metabolic dependencies in a tumor and context-dependent manner: (1) Aerobic glycolysis, controlled by MYC; (2) OxPhos, fuelled by different microenvironmental substrates and controlled by Imp2 or PGC-1α; (3) Lipid metabolism, increasing either fatty acid synthesis and storage in lipid droplets (LDs) or utilization via mitochondrial FAO; (4) CSCs can be dependent on alternative substrates and pathways such as aminoacids, ketone bodies, PPP or purines. Interestingly, the metabolic phenotypes described for CSCs ensure the maintenance of cellular redox state. Keeping redox balance is crucial for CSCs in order to maintain their stemness characteristics, differentiation ability and resistance to chemo and radiotherapy, constituting one of the most important vulnerabilities independently of their origin or cellular context. ROS – reactive oxygen species, OxPhos – oxidative phosphorylation, LDs – lipid droplets, PPP – pentose phosphate pathway, FAO – fatty acid oxidation, TCA – tricarboxylic acid cycle.

Over the last few years, a huge collective effort to decipher metabolic reprogramming occurring in cancer has taken place. Technical advances have allowed the determination of the great metabolic heterogeneity, not only among individuals suffering from one type of cancer, but within a single tumor. Collectively, present literature indicates that both metabolic and redox state diversity define CSCs phenotype and fate, determining response to therapy. Importantly, most of the metabolic dependencies described for CSCs in diverse tumor entities have the tight control of redox state as a common factor (Figure 3), unveiling an important vulnerability that could provide new therapeutic opportunities. Other factors, such as tumor metabolic heterogeneity, microenvironmental cues or a cross-talk through metabolic and redox signaling between CSCs and cancer cells or stromal components (Riemann et al., 2011; Chen X. et al., 2016; Chang et al., 2018; Luo et al., 2018) can play an additional role in cancer progression and chemoresistance. Therefore, current knowledge suggests that carefully designed therapies, which target metabolically diverse populations and consider the tumor microenvironment may be crucial in order to develop more effective metabolism-focused treatment strategies.

## Author Contributions

PJ, BdL-D, and BP-A designed the figures. PS developed the study concept, obtained funding and wrote the final version of the manuscript. All authors contributed to conception and design of the manuscript, wrote sections, revised, read and approved the submitted version.

## Conflict of Interest Statement

The authors declare that the research was conducted in the absence of any commercial or financial relationships that could be construed as a potential conflict of interest.

## Abbreviations

α-ADD: alpha-aminoadipate
2DG: 2-deoxy-D-glucose
AML: acute myeloid leukemia
ALDH1: aldehyde dehydrogenase 1
ATO: arsenic trioxide
BET: bromodomain and extra-terminal motif
BSO: buthionine sulfoximide
CAFs: cancer associated fibroblast
CML: chronic myeloid leukemia
CSCs: cancer stem cells
DHA: docosahexaenoic acid
DOX: doxorubicin
DQA: dequalinium
DRP-1: dynamin-related protein 1
EGCG: epigallocatechin gallate
EPA: eicosapentaenoic acid
ERRα: estrogen-related receptor alpha
EMT: epithelial-mesenchymal transition
ETC: electron transport chain
FAs: fatty acids
FAO: fatty acid oxidation
FASN: fatty synthase
FOXO: forkhead box
G6P: glucose-6-phosphate
GPx: glutathione peroxidase
HBP: hexosamine biosynthetic pathway
LDs: lipid droplets
LPA: lysophosphatidic acid
MAO-B: monoamine oxidase-B inhibitor
MCT 1/2: monocarboxylate transporter 1 and 2
Mdivi-1: mitochondrial division inhibitor
MPP: mitochondria penetrating peptide
mtDNA: mitochondrial DNA
mTOR: mammalian target of rapamycin
MTS: mitochondria targeting sequences
MUFAs: monounsaturated fatty acids
NER: nucleotide excision repair
NRF2: nuclear factor erythroid 2–related factor 2
OxPhos: oxidative phosphorylation
PDAC: pancreatic ductal adenocarcinoma
PDX: patient-derived xenograft
PEITCs: phenethyl isothiocyanates
PGC-1α: peroxisome proliferator-activated receptor gamma coactivator 1-alpha
PHGDH: phosphoglycerate dehydrogenase
PI3K: phosphoinositide 3-kinase
PPP: pentose phosphate pathway
ROS: reactive oxygen species
S1P: sphingosine-1-P
SOD: superoxide dismutase
SREBP1: sterol regulatory element-binding protein 1
SCD-1: stearoyl-CoA desaturase
TCA: tricarboxylic acid
TICs: tumor initiating cells
TNBC: triple negative breast cancer
TPP: triphenylphosphonium
